# Starting an All-Oral Longer Regimen in a Primary Multidrug-Resistant Pulmonary Tuberculosis Patient at a District Tuberculosis Center for the First Time: A Rare Case

**DOI:** 10.7759/cureus.27146

**Published:** 2022-07-22

**Authors:** Sankalp Yadav

**Affiliations:** 1 Medicine, Shri Madan Lal Khurana Chest Clinic, New Delhi, IND

**Keywords:** mycobacterium tuberculosis, drug resistance, multidrug-resistant, bedaquiline, covid-19, tuberculosis

## Abstract

Tuberculosis (TB) is a disease known to mankind for ages. The situation due to this infection in low- and middle-income countries is grave. The coronavirus disease 2019 (COVID-19) pandemic has only added up to the woes. The situation is alarming due to the isolation of drug-resistant *Mycobacterium* strains in patients with no history of TB. With the inclusion of new drugs for the management of TB, such as bedaquiline (Bdq), prompt diagnosis and management are feasible. The author herein presents the first case of a primary multidrug-resistant pulmonary TB patient managed on an all-oral longer regimen with Bdq started at a district TB center (DTC) for the first time in the pandemic of COVID-19. This case is unique as during the COVID-19 pandemic, healthcare facilities were saturated, and thus starting treatment after admission was very difficult. Also, the chances of cross-infection in TB patients were present due to weak immunity. This case is very important as this novel management at a DTC would help immensely in resource-limited countries where hospital admissions are difficult due to the COVID-19 pandemic and the burden of TB is very high.

## Introduction

Tuberculosis (TB) is a significant public health issue [1]. The disease is known to be a considerable cause of morbidity and mortality in low-income countries [2]. In resource-limited countries where the healthcare systems are already overwhelmed by the current coronavirus disease 2019 (COVID-19) pandemic, the prevalence of TB is just adding up to the agony [3]. These countries were already having a large number of TB cases and the lockdowns during the current pandemic of COVID-19 had resulted in a very high number of TB cases [4]. The possible causes could be longer close contacts, altered immunity, improper ventilation in residence, lung inflammation, stress due to COVID-19, use of steroids for COVID-19 management, lockdowns, and worsening of blood sugar control. Other contributors could be poverty and a lack of financial stability along with malnutrition, substance misuse, poor housing conditions, and HIV/AIDS incidence. Furthermore, due to oversaturated health facilities inclined toward COVID-19 care, patients with other diseases like TB were neglected.

Drug-resistant TB (DR-TB) could be either multidrug-resistant TB (MDR-TB), isoniazid mono-resistance, extensively drug-resistant TB (XDR-TB), or pre-extensively drug-resistant TB (pre-XDR-TB) [5]. MDR-TB is defined as a disease caused by *Mycobacterium tuberculosis* (MTb) complex strains with resistance to, at least, isoniazid and rifampicin [5]. India has the second-highest load of MDR-TB in the world with 1.3 million DR-TB cases in 2018 [6,7]. The prevalence of MDR-TB in new cases is 2.8% and 12% in previously treated cases [7]. The incidence of MDR-TB/rifampicin-resistant TB (RR-TB) was 135,000 [7]. The management of DR-TB in India is through the Programmatic Management of Drug-Resistant TB (PMDT), which is a very detailed document that emphasizes the decentralization of the diagnosis and treatment of MDR-TB at the district level [7]. The author herein presents the first case in the world of primary pulmonary MDR-TB in an Indian male who was not eligible for the new shorter MDR-TB treatment and thus was started on an all-oral longer regimen with bedaquiline (Bdq) and an optimized background regimen (OBR) for the first time at a very large and high-burden district TB center (DTC) in the western part of the national capital of India.

## Case presentation

A 20-year-old Indian male of the low socioeconomic group visited our outpatient department (OPD) with principal complaints of fever with night sweats, chest pain, cough, breathlessness, weight loss, and weakness for three weeks. The fever was initially (three days) of low grade and then high grade in the evening with chills and no rigors and was relieved (usually for three to four hours) after taking paracetamol. The chest pain was intermittent, bilateral on the upper and middle chest wall, and aggravated on exertion or coughing. The cough was continuous and was associated with a thick, clear, non-foul-smelling expectoration. He also had breathlessness on exertion, which aggravated during cough episodes, and was relieved after taking an antitussive. He reported weakness and weight loss of 6 kg in the last month. There was no hemoptysis, seizure, or loss of taste and smell. There was no known contact with a TB or COVID-19 patient in the recent past, and there was no history of any similar complaints in the family. There was no history of substance abuse or drug allergy.

The general examination was suggestive of a lean individual weighing 49 kg with normal vitals. There was no pallor, icterus, cyanosis, lymphadenopathy, or edema. On systemic examination, a dull note was heard on bilateral upper lobes, and auscultation revealed reduced breath sounds in the left and right upper zones of the chest. The rest of the systemic examinations were unremarkable.

Based on the initial findings, he was referred to the lab for sputum for acid-fast bacillus (AFB) test under a fluorescent microscope using rhodamine auramine stain, which turned out as sputum positive (+2). Subsequently, a cartridge-based nucleic acid amplification test (CBNAAT) was done, which was suggestive of MTb detected medium with rifampicin resistance.

Based on these reports, he was declared a case of primary pulmonary MDR-TB and was planned for a pre-treatment evaluation for initiating the shorter MDR-TB treatment as per the PMDT guidelines along with other formalities like sending the sample for line probe assay (LPA), culture, and drug susceptibility testing (DST). The details of the pre-treatment evaluation are mentioned in Table 1.

**Table 1:** Laboratory investigations

Laboratory investigation	Patient value	Reference value
Hemoglobin	11 g/dL	11.9-15
Total leukocyte count	7.2 × 10^9^/L	4-10
Platelets	3.5 x 10^9^/L	1.5-4.0 x 10^9^
Glucose (fasting)	4.17 mmol/L	3.9-5.6
Bilirubin (conjugated)	0.7 µmol/L	<1 mg/dL
Creatinine	57 µmol/L	53-97.2
Uric acid	177 µmol/L	130-378

**Figure 1:**
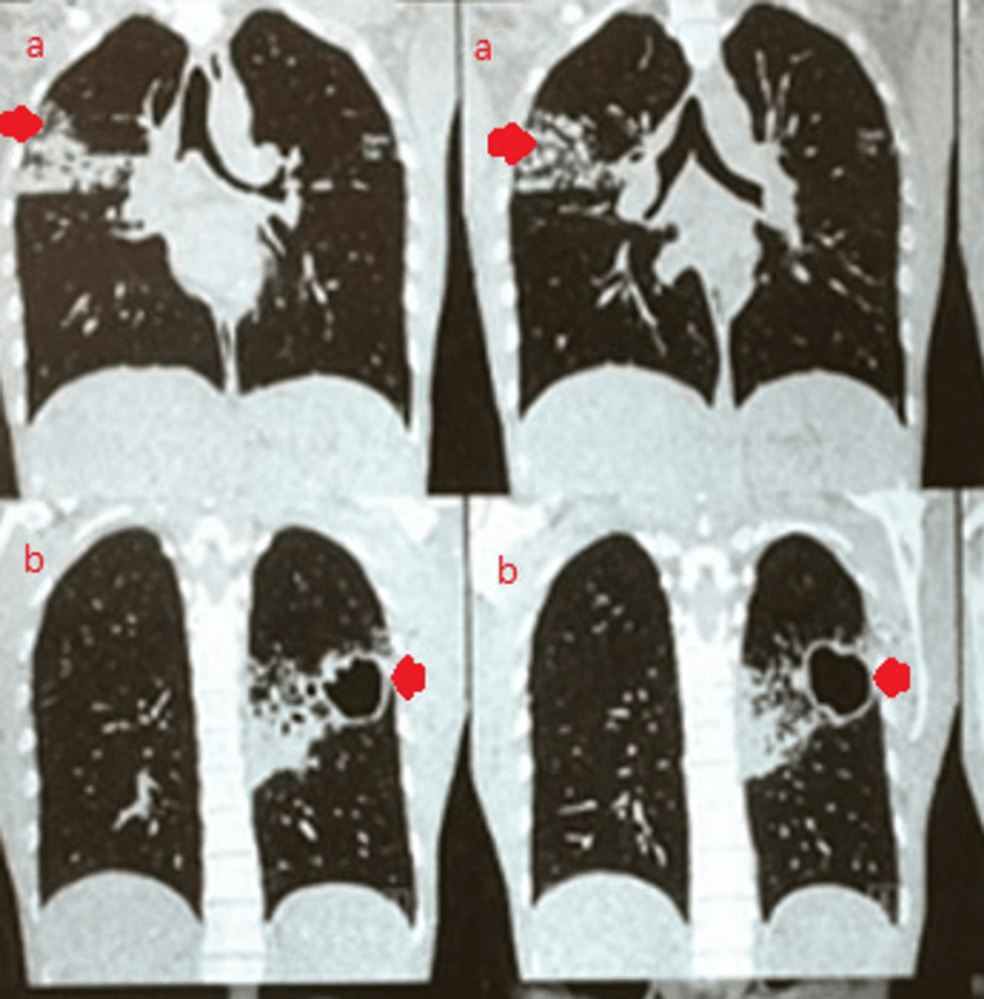
High-resolution computed tomography (HRCT) of the chest showing multiple coalescing cavitary lesions in the left upper lobe with peri-regional nodular and alveolar opacities and multiple coalescing nodular and alveolar opacities in the right upper lobe. (A) Multiple coalescing and alveolar opacities in the right upper lobe. (B) Multiple coalescing cavitary lesions in the left upper lobe with peri-regional and alveolar opacities.

Urine (routine/microscopic) and thyroid profile were normal. HIV status was non-reactive. Chest X-ray (posteroanterior view) showed bilateral upper-zone consolidation with a thick-walled cavity in the left para-hilar region. A high-resolution computed tomography (HRCT) of the chest was performed, which showed multiple coalescing cavitary lesions in the left upper lobe with peri-regional nodular and alveolar opacities, and on the right upper lobe, it showed multiple coalescing nodular and alveolar opacities (Figure 1). A few subcentimetric mediastinal nodes were also seen. The sputum AFB culture revealed resistance to both isoniazid and rifampicin.

The audiogram was suggestive of bilateral sensory neural hearing loss (SNHL) at high frequencies. Therefore, considering the age of the patient and SNHL on an audiogram and after taking an opinion at the nodal TB center, it was decided to start him on an all-oral longer regimen with drugs like Bdq. Thus, he was advised of further tests, including ECG and serum electrolytes. These reports were within normal limits with a Fridericia's corrected QT interval (QTcF) of 368 ms on ECG.

An all-oral longer MDR-TB regimen is of 18-20 months with no discrete intensive and continuation phases [8]. New drugs like Bdq and delamanid would be used for six months duration while the dose of linezolid will be tapered to 300 mg after the initial six to eight months of treatment [8]. This patient was started on this all-oral longer regimen from our DTC for the first time with Bdq 400 mg for two weeks followed by 200 mg for 22 weeks, with an OBR consisting of levofloxacin 1000 mg, linezolid 600 mg, cycloserine 750 mg, clofazimine 100 mg, and pyridoxine 100 mg. In the past, all such cases were started at the nodal DR-TB center, and, usually, patients were admitted for initial two weeks [2]. Currently, the patient is on this regimen for 15 months and is stable with no remarkable adverse drug reactions. He was transferred to his native village at his request. Besides, he was regularly counseled and followed up in the OPD before his transfer.

## Discussion

The decentralization of management of DR-TB cases to DTC is a notable change for the success of PMDT in the National Tuberculosis Elimination Program (NTEP) [9]. This change has not only increased the accessibility of the NTEP to the masses but also has helped in reducing the load of heavily burdened nodal TB centers [9]. With the ongoing pandemic of COVID-19, there is a saturation of healthcare centers with the staff having multiple psychological and behavioral impacts due to COVID-19, resulting in a lack of proper attention to the already existing diseases like TB [10]. As stated earlier, the prevalence of MDR-TB in new cases is 2.8% and reports of primary DR-TB are scarce and are limited to a few case reports. However, in resource-limited countries with a high load of TB, DR-TB should always be in the mind of treating clinicians [2]. There are isolated reports of primary MDR-TB in cases of breast, lymphadenitis, sternum, and pleural effusion. However, to date, no reports of starting an all-oral longer regimen with Bdq in a primary multidrug-resistant pulmonary TB patient at a DTC are reported. All these cases were started at the nodal DR-TB centers where they were admitted and were under close observation for any adverse drug reactions.

This case thus becomes an extremely important case clinically as it would help in the initiation of treatment of DR-TB cases at the nearest DTC, thereby reducing the time of initiation, finances incurred due to traveling, and probably would also help in higher cure rates as patients are more comfortable to report to their nearby DTC rather than any far-off nodal DR-TB center [9]. Besides, TB is most prevalent in low- to middle-income countries. As a result, it becomes very imperative that DR-TB cases should be started on DTCs. Not only this will increase the chances of a higher rate of treatment completion but also an early recording and reporting of any adverse drug reaction with a reduced number of lost to follow-up cases. Again, in the time of the COVID-19 pandemic, this becomes even more essential that all the DR-TB cases should be started on the DTC as this will help in decreasing the burden on the overwhelmed health facilities, as there is a shortage of resources in some of the high-burden countries where infrastructure and other shortfalls have a stark impact on the overall TB control efforts. Again, in hilly and other difficult terrains, such initiations at the nearby DTC would only help in achieving the desired results of prompt diagnosis and management of DR-TB.

## Conclusions

This case will serve as an important tool in the management of MDR-TB, where due to issues like a deranged audiogram, age, or any other problem in the PTE, a patient is not eligible for the shorter MDR-TB regime. In the present situation, where the viral pandemic is in full swing, the diagnosis and management of rare presentations of common diseases is a welcome addition to the medical literature. Besides, large-scale studies, especially in high TB burden areas, to determine the extent of primary drug resistance is the need of the hour.
